# Ventricular apparent diffusion coefficient measurements in patients with neoplastic leptomeningeal disease

**DOI:** 10.1186/s40644-020-00305-2

**Published:** 2020-06-29

**Authors:** James M. Debnam, Ryan B. Said, Heng-Hsiao Liu, Jia Sun, Jihong Wang, Wei Wei, Dima Suki, Rory R. Mayer, T. Linda Chi, Leena Ketonen, Nandita Guha-Thakurta, Jeffrey S. Weinberg

**Affiliations:** 1 Department of Neuroradiology, The University of Texas MD Anderson Cancer Center, 1400 Pressler Blvd., Unit 1482, Houston, TX 77030 USA; 2 Department of Biostatistics, The University of Texas MD Anderson Cancer Center, Houston, TX USA; 3 Department of Radiation Physics, The University of Texas MD Anderson Cancer Center, Houston, TX USA; 4 Department of Neurosurgery, The University of Texas MD Anderson Cancer Center, Houston, TX USA; 5 Department of Neurosurgery, University of California San Francisco, San Francisco, CA USA

**Keywords:** Apparent diffusion coefficient, Ventricles, Cerebrospinal fluid, Magnetic resonance imaging

## Abstract

**Background:**

To test the hypothesis that intraventricular ADC values can be used to determine the presence of neoplastic leptomeningeal disease (LMD).

**Materials and methods:**

ADC values were measured at multiple sites in the ventricular system in 32 patients with cytologically-proven LMD and 40 control subjects. Multiple linear regression analysis was used to determine the mean difference of ADCs between the LMD and control groups after adjusting for ventricle size and tumor type. Receiver operating characteristics (ROC) analysis was performed and optimal ADC value cut-off point for predicting the presence of LMD. ADC was compared to T1 enhancement and FLAIR signal hyperintensity for determining the presence of LMD.

**Results:**

After adjusting for ventricular volume and tumor type, the mid body of lateral ventricles showed no significant difference in ventricular volume and a significant difference in ADC values between the control and LMD groups (*p* > 0.05). In the mid-body of the right lateral ventricle the AUC was 0.69 (95% CI 0.57–0.81) with an optimal ADC cut off point of 3.22 × 10^− 9^ m^2^/s (sensitivity, specificity; 0.72, 0.68). In the mid-body of left lateral ventricle the AUC was 0.7 (95% CI 0.58–0.82) with an optimal cut-off point of 3.23 × 10^− 9^ m^2^/s (0.81, 0.62). Using an average value of HU measurements in the lateral ventricles the AUC was 0.73 (95% CI 0.61–0.84) with an optimal cut off point was 3.11 × 10^− 9^ m^2^/s (0.78, 0.65). Compared to the T1 post-contrast series, ADC was predictive of the presence of LMD in the mid-body of the left lateral ventricle (*p* = 0.036).

**Conclusion:**

Complex interactions affect ADC measurements in patients with LMD. ADC values in the lateral ventricles may provide non-invasive clues to the presence of LMD.

## Introduction

Leptomeningeal disease (LMD) is the dissemination of cancer cells throughout the leptomeningeal space and portends a dismal prognosis with increased mortality rates [1–7]. Rarely seen only a few decades ago, the incidence of LMD has been increasing since then, now occurring in 3–8% of patients with cancer, [1–3] likely related to improved survival and advances in imaging techniques.

The National Comprehensive Cancer Network requires one of the following three criteria to diagnose LMD: (1) tumorous cells in the CSF on cytological evaluation, (2) clinical and CSF laboratory findings in keeping with LMD (elevated protein and white blood cell counts and low glucose level) in patients with a cancer, or (3) demonstration of LMD on radiological studies regardless of the clinical examination [8]. Although the diagnosis of LMD via cerebrospinal fluid (CSF) cytology is the gold standard, it is invasive and is only 80–95% sensitive [4]. MRI is also a valuable adjunct and alternative to repeated large-volume CSF analysis [5, 6]. Gadolinium-enhanced T1-weighted and post-contrast FLAIR MRI remain the most sensitive imaging techniques for diagnosing LMD [3, 7, 9] as tumor cells adhere to the leptomeninges [10–12]. However, in the literature the sensitivity of MRI in this setting ranges between 53 and 79% [13–18].

Diffusion-weighted imaging is a method with wide applications in stroke and tumor imaging [19–21]. In this procedure, the magnitude of fluid movement within a voxel is measured according to the apparent diffusion coefficient (ADC). A low ADC value indicates decreased molecular movement in the tissue sample, whereas a high ADC value indicates freer diffusion.

Radioisotope CSF flow studies have demonstrated compartmentalization of CSF in patients with LMD [22–26]. Thus, we hypothesized that compartmentalization of CSF will restrict movement of CSF and lead to decreased ADC values. Potentially, measuring ADC values could be a non-invasive technique to predict the presence of LMD in patients with cancer. Therefore, the purpose of this study was to measure and compare the ADC values in the ventricular system in patients with active cancer and LMD compared to a control group of patients with a prior history of cancer who are clinically NED (no evidence of disease).

## Materials and methods

### Subjects

The study was approved by our Institutional Review Board who waived the requirement for informed consent. Data acquisition was performed in compliance with all applicable Health Insurance Portability and Accountability Act regulations. Clinical data and imaging studies were reviewed to identify patients with histological diagnoses of breast cancer or lymphoma via lumbar puncture who underwent conventional MRI of the brain with ADC maps. Patient were included in the study group if they had an MRI examination of the brain with T1 post-contrast and FLAIR sequences, and cytological evidence of LMD. Patients in the control group had a similar MR examination of the brain, available cytological evaluation of CSF has to be negative for disease involvement, and the patients were clinically asymptomatic classifying them as NED.

### Imaging protocol

MRI was performed following our institutional protocol which may have slightly varied during the study timeframe. MRI studies of the brain were performed on 1.5 T scanners (GE Healthcare, Milwaukee, Wisconsin). Typical examinations included contrast-enhanced axial T1-weighted spin-echo (TR/TE, 850/14–22) and FLAIR (TR/TE, 10000/147) imaging. Axial DTI (TR/TE, 11400/8–15) was performed with a B value of 0 & 1200 (27–32 directions) with a slice thickness of 3.5 mm and an acquisition time of approximately 5 min. Diffusion tensor images were analyzed using the FuncTool software program (version 4.5.3) on an AW workstation (version 4.4; both from GE Healthcare). ADC was defined using the formula $ADC=\frac{\lambda_1+{\lambda}_2+{\lambda}_3}{3}$, in which λ_1,_ λ_2_, and λ_3_ are the 3 eigenvalues calculated using diffusion tensor images.

**Fig. 1:**
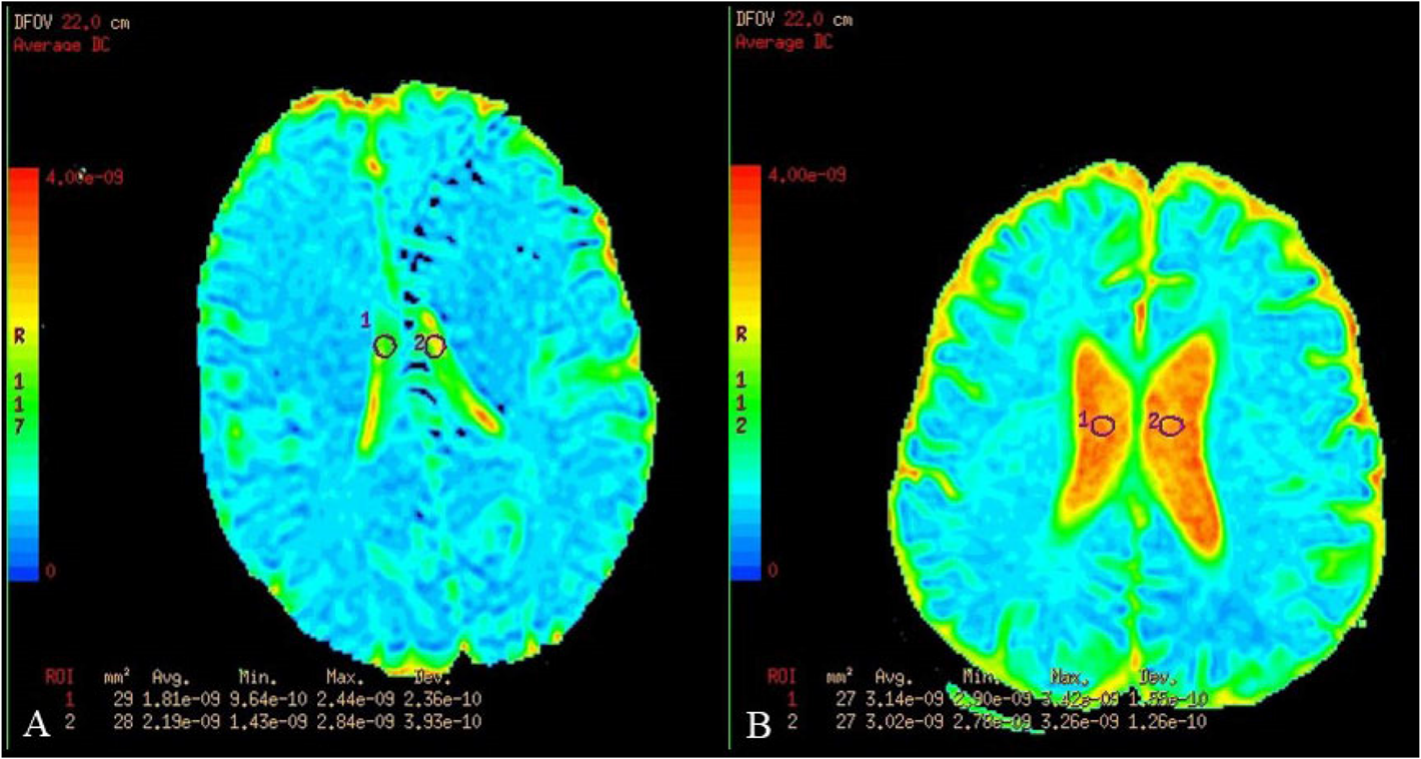
ROI placement in the mid body of the lateral ventricles of the control groups. **a**, Breast cancer control. ADC values were 1.81 × 10^− 9^ mm^2^/s and 2.19 × 10^− 9^ mm^2^/s on the right and left side, respectively. The volume of the lateral ventricles was 7.1 cm^3^. **b**, ADC values were 3.14 × 10^− 9^ mm^2^/s and 3.02 × 10^− 9^ mm^2^/s on the right and left side, respectively. The volume of the lateral ventricles was 53.3 cm^3^

**Fig. 2:**
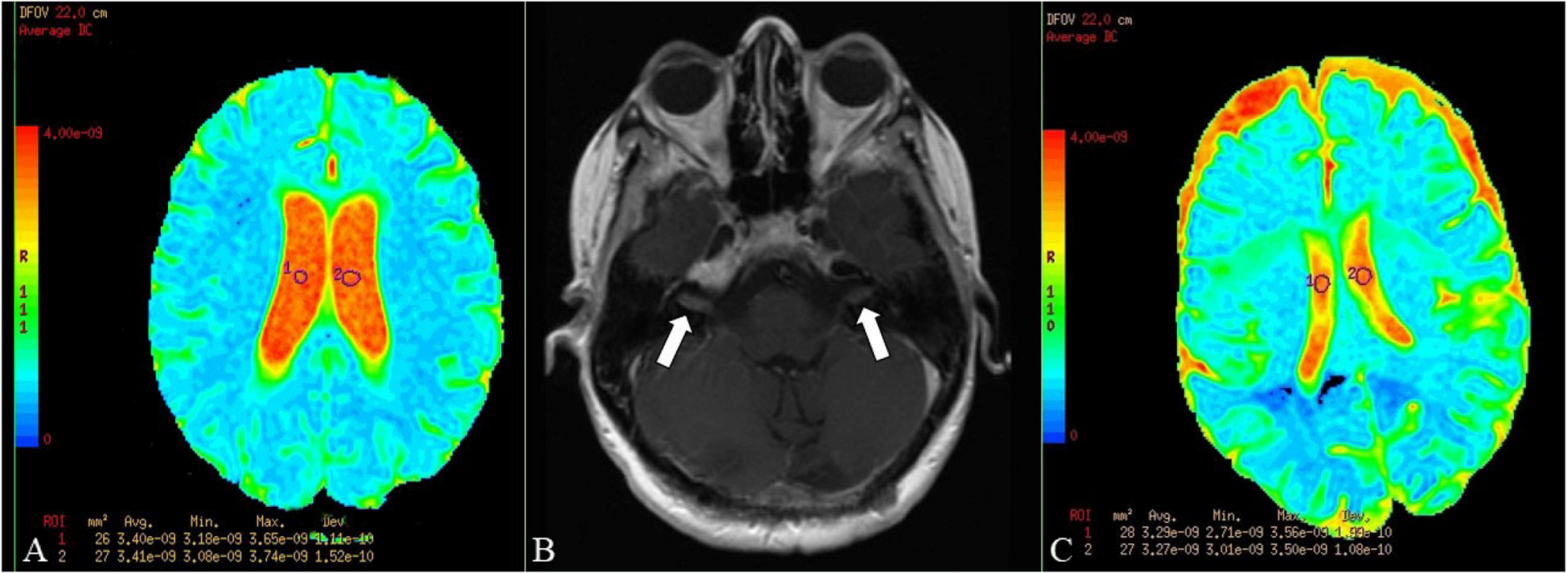
ROI placement in the mid body of the lateral ventricles in patients with LMD. **a**, Breast cancer with LMD. ADC values were 3.4 × 10^− 9^ m^2^/s and 3.41 × 10^− 9^ m^2^/s on the right and left side, respectively. The volume of the lateral ventricles was 60.4 cm^3^. **b**, Axial T1 post-image series. LMD characterized by diffuse enhancement in the bilateral internal auditory canals (arrows). **c**, Lymphoma with LMD. ADC values were 3.29 × 10^− 9^ m^2^/s and 3.27 × 10^− 9^ m^2^/s on the right and left side, respectively. The volume of the lateral ventricles was 13.4 cm^3^. No abnormalities were present on the T1 post-contrast or FLAIR series (not shown)

We hypothesized that multiple complex interactions may affect HU values in the ventricular system including but not limited to various diameters of different components of the ventricular system, pressure differences, CSF flow rate and eddy formation. Therefore, to determine if there is a single or multiple sites where ventricular ADC values are different between the study and control groups, ROIs were independently drawn manually on ADC maps in the frontal horns, mid-body, and atria of the lateral ventricles bilaterally, and in the 3rd and 4th ventricles for both the control and study groups by a neuroradiologist (JMD) and a radiology resident (RBS) who were not blinded as the presence or absence of active disease (Figs. 1 and 2). All ADC values were automatically calculated and expressed in m^2^/s. To minimize partial volume effects, axial slices were localized for analysis by identifying regions for which the slices were present immediately superior and inferior to site where the ROI was placed. Care was taken to ensure that the ROIs in all scans was between 25 and 30 mm^2^ and an effort was made to exclude the choroid plexus from the measured sites.

To determine if ventricular volume affects the ADC values, the volume of the lateral, 3rd and 4th ventricles were measured by consensus using imaging biomarker explorer (IBEX) software that was developed “in-house” and enabled us to measure the ventricular volumes [27]. For comparison in both the LMD and control groups, the presence of leptomeningeal enhancement and FLAIR signal hyperintensity on conventional MR imaging were recorded as being present or absent.

### Statistical analysis

Intraclass correlation coefficient (ICC) with 95% confidence intervals (CI) was used to evaluate the inter-reader agreement of the ADC measurements. The ICCs for all observations for each subsite were calculated using the two-way mixed effect model (ICC [C,1]).

ADC measurements from the two reviewers were averaged for data analysis. Descriptive statistics for ADC, such as mean, standard deviation (SD), minimum, median, and maximum, by subsite and tumor type were calculated. First, univariate analysis was conducted to compare ADC values and ventricular volumes between the LMD and control groups by two-sample t-test. Second, multiple linear regression was used to determine the mean difference of ADCs between the LMD and control groups after adjusting for ventricle size and tumor type. The regression equation is presented as:

$$ {y}_{ADC}={b}_0+{b}_1\ast {x}_{ventricle\ size}+{b}_2\ast {x}_{Tumor\ type}+{b}_3\ast {x}_{LMD/ control\ group} $$

These estimates determine the relationship between the independent variables (the ventricle size, tumor type, and LMD/control group) and the dependent variable (ADC). Third, scatter plots were generated at each site in the ventricular system to compare the adjusted lateral ventricular volume to the ADC values.

Fourth, to determine if ventricular ADC values could predict the presence of LMD, receiver operating characteristics (ROC) analysis was performed to determine the area under the ROC curve and optimal ADC value cut-off point in predicting the presence of LMD with associated sensitivity and specificity. ROC analysis was also performed on the average HU value from each of the 6 measured site in the lateral ventricles.

Finally, the Fisher exact test was used to compare the presence of T1 enhancement and sulcal FLAIR signal hyperintensity in the breast cancer and lymphoma LMD and controls groups. Multiple logistic regressions were used to determine if the presence of LMD on conventional MRI, as characterized by T1 enhancement or sulcal FLAIR signal hyperintensity, could be predicted by the ADC values in the ventricles. All tests were two-sided and *p*-values of 0.05 or less were considered statistically significant. Statistical analysis was carried out using SAS version 9.4 (SAS Institute, Cary, NC).

## Results

Seventeen consecutive patients with breast cancer and LMD (15 women and 2 men, age range 32–70 years, mean 49.5 ± 10.6 years) and 15 consecutive patients with lymphoma and LMD (10 men and 5 women, 29–81, mean 56.4 ± 16.5 years) confirmed via cytological evaluation formed the study group. One outlier patient in the lymphoma LMD study group was excluded due to an excessively large ventricular size. The control group comprised 20 consecutive patients with a prior history of breast cancer (19 women and 1 man; 31–68, 52 ± 7.8 years,) and 20 consecutive patients with a prior history of lymphoma (11 men and 9 women; age 3–71 53.2 ± 20.2 years) who were clinically NED.

The ICC between the 2 reviewers for the measurement of ADC values at all sites were ≥ 0.81 (excellent) (Table 1).

**Table 1:** ADC measurement inter-observer variability

ADC location	ICC	ICC 95% CI
Right frontal horn	0.97	0.96–0.98
Left frontal horn	0.81	0.71–0.88
Right lateral ventricle	0.93	0.89–0.95
Left lateral ventricle	0.89	0.84–0.93
Right atrium	0.83	0.74–0.89
Left atrium	0.97	0.96–0.98
3rd ventricle	0.98	0.96–0.99
4th ventricle	0.95	0.93–0.97

< 0.40, poor; 0.41–0.60, moderate

0.61–0.80, good; ≥0.81, excellent

### Comparison of ADC values and ventricular volumes

The average ADC values of the reviewers comparing the control and LMD group are provided in Table 2. Without adjusting for the ventricular volumes, a significant difference in ADC values was noted between the breast cancer control and breast cancer LMD groups in the following sites: right frontal horn (*p* <  0.014), left frontal horn (*p* <  0.044), right mid-body (*p* < 0.033), right atrium (*p* <  0.004), left atrium (*p* <  0.043), and third ventricle (*p* <  0.013). For the patients with lymphoma a significant difference in ADC values between control and LMD groups was present in the left frontal horn (p <  0.044), left mid-body (*p* <  0.46) and third ventricle (*p* < 0.018). When averaging the ADC values in the lateral ventricles, there was a significant difference between the breast cancer group and control group (*p* = 0.006) while the lymphoma group approached significance (*p* = 0.052). Ventricular volume measurements are provided in Table 3. For the breast cancer groups there was a significantly larger size of the lateral ventricles (*p* = 0.005) and the total ventricular volume (*p* = 0.004) in the LMD group compared to controls. No significant difference was noted between ventricular sizes in the lymphoma LMD versus control group (*p* > 0.05).

**Table 2:** Comparison of ADC values between LMD and control groups at various sites in the ventricular system

Site	Disease		control	LMD	Total	*p* value
Right frontal horn	Breast	N	20	17	37	0.014
		Mean (SD)	2.55 (0.73)	3.06 (0.36)	2.79 (0.64)	
	Lymphoma	N	20	15	35	0.051
		Mean (SD)	2.76 (0.69)	3.14 (0.25)	2.92 (0.57)	
Left frontal horn	Breast	N	20	17	37	0.044
		Mean (SD)	2.79 (0.56)	3.11 (0.35)	2.94 (0.50)	
	Lymphoma	N	20	15	35	0.071
		Mean (SD)	2.95 (0.46)	3.17 (0.13)	3.04 (0.37)	
Right mid body	Breast	N	20	17	37	0.033
		Mean (SD)	3.04 (0.38)	3.26 (0.13)	3.14 (0.31)	
	Lymphoma	N	20	15	35	0.122
		Mean (SD)	3.18 (0.21)	3.28 (0.11)	3.22 (0.18)	
Left mid body	Breast	N	20	17	37	0.104
		Mean (SD)	3.15 (0.29)	3.28 (0.11)	3.21 (0.23)	
	Lymphoma	N	20	15	35	0.046
		Mean (SD)	3.19 (0.19)	3.30 (0.07)	3.24 (0.16)	
Right atrium	Breast	N	20	17	37	0.004
		Mean (SD)	2.70 (0.48)	3.10 (0.25)	2.89 (0.43)	
	Lymphoma	N	20	15	35	0.25
		Mean (SD)	3.07 (0.36)	3.21 (0.32)	3.13 (0.34)	
Left atrium	Breast	N	20	17	37	0.043
		Mean (SD)	2.81 (0.45)	3.09 (0.34)	2.94 (0.42)	
	Lymphoma	N	20	15	35	0.628
		Mean (SD)	3.24 (0.26)	3.28 (0.23)	3.26 (0.24)	
3rd ventricle	Breast	Number	20	17	37	0.013
		Mean (SD)	2.72 (0.54)	3.15 (0.45)	2.92 (0.54)	
	Lymphoma	N	20	15	35	0.018
		Mean (SD)	3.16 (0.36)	3.42 (0.19)	3.27 (0.33)	
4th ventricle	Breast	N	20	17	37	0.452
		Mean (SD)	3.39 (0.23)	3.46 (0.34)	3.42 (0.29)	
	Lymphoma	N	20	15	35	0.079
		Mean (SD)	3.38 (0.31)	3.54 (0.17)	3.45 (0.27)	
Lateral ventricle (average)	Breast	N	20	17	37	0.006
		Mean (SD)	2.84 (0.39)	3.15 (0.21)	2.98 (0.35)	
	Lymphoma	N	20	15	35	0.052
		Mean (SD)	3.07 (0.29)	3.23 (0.14)	3.14 (0.25)	

*N* Number

*SD* standard deviation

**Table 3:** Comparison of ventricular volumes between control and LMD groups

Site	Disease		control	LMD	Total	*p* value
Lateral ventricles	Breast	N	20	17	37	0.005
		Mean (SD)	18.06 (8.40)	30.29 (15.64)	23.68 (13.57)	
	Lymphoma	N	20	15	35	0.454
		Mean (SD)	1.85 (1.05)	2.10 (0.80)	1.96 (0.95)	
3rd ventricle	Breast	N	20	17	37	0.052
		Mean (SD)	0.91 (0.37)	1.98 (2.35)	1.40 (1.68)	
	Lymphoma	N	20	15	35	0.454
		Mean (SD)	1.85 (1.05)	2.10 (0.80)	1.96 (0.95)	
4th ventricle	Breast	N	20	17	37	0.663
		Mean (SD)	1.01 (0.30)	1.09 (0.75)	1.05 (0.55)	
	Lymphoma	N	20	15	35	0.925
		Mean (SD)	1.14 (0.50)	1.13 (0.40)	1.14 (0.45)	
Total volume	Breast	N	20	17	37	0.004
		Mean (SD)	19.98 (8.73)	33.37 (17.22)	26.13 (14.76)	
	Lymphoma	N	20	15	35	0.568
		Mean (SD)	36.90 (19.59)	33.40 (14.90)	35.40 (17.58)	

*N* Number

*SD* standard deviation

### Difference of ADC values after adjusting for ventricle size and tumor type

After adjusting for ventricular volume and tumor type, a significant difference in ventricular volume between the control and LMD groups was found at all sites except for left (*p* > 0.058) mid-body of the lateral ventricles, although this sits approached a significant difference. When comparing the ADC values between the patients with breast cancer and lymphoma who had LMD, a significant difference was found in the right (*p* < 0.048) and left (*p* < 0.001) atrium and in the third ventricles (*p* < 0.002) with the lymphoma group having higher ADC values. When comparing ADC values between the control groups and the patients with LMD, a significant difference was noted between the ADC values at all sites except for the left atrium (*p* > 0.09) and fourth ventricle (*p* > 0.068). When averaging the HU values in the lateral ventricles, a significant difference was found in ventricular size between the control and LMD groups (*p* < 0.001). No significant difference was found between the ADC values of the breast cancer and lymphoma groups (*p* = 0.18) but there was a difference in ADC values between the control and LMD groups (*p* = 0.001). These results are summarized in Table 4.

**Table 4:** Difference of ADC values after adjusting for ventricle size and tumor type

Site	Covariate	Beta	95% CI	*p* value
Right frontal horn	Volume	0.021	0.013, 0.028	< 0.001
	Tumor	−0.03	−0.268, 0.201	0.775
	Group LMD	0.352	0.123, 0.581	0.003
Left frontal horn	Volume	0.01	0.004, 0.016	0.002
	Tumor	0.027	−0.166, 0.22	0.779
	Group LMD	0.233	0.045, 0.422	0.016
Right mid body	Volume	0.004	0, 0.008	0.036
	Tumor	0.051	−0.065, 0.167	0.382
	Group LMD	0.138	0.025, 0.252	0.018
Left mid body	Volume	0.003	0, 0.006	0.058
	Tumor	0.012	−0.081, 0.104	0.805
	Group LMD	0.103	0.013, 0.194	0.026
Right atrium	Volume	0.009	0.004, 0.015	0.001
	Tumor	0.171	0.002, 0.341	0.048
	Group LMD	0.103	0.013, 0.194	0.026
Left atrium	Volume	0.007	0.001, 0.012	0.013
	Tumor	0.271	0.11, 0.432	0.001
	Group LMD	0.135	−0.022, 0.292	0.09
3rd ventricle	Volume	0.121	0.052, 0.191	< 0.001
	Tumor	0.297	0.111, 0.483	0.002
	Group LMD	0.268	0.079, 0.457	0.006
4th ventricle	Volume	0.264	0.15, 0.378	< 0.001
	Tumor	0.012	−0.102, 0.126	0.832
	Group LMD	0.106	−0.008, 0.22	0.068
Lateral average	Volume	0.009	0.005, 0.013	< 0.001
	Tumor	0.083	−0.039, 0.205	0.18
	Group LMD	0.198	0.079, 0.318	0.001

**Fig. 3:**
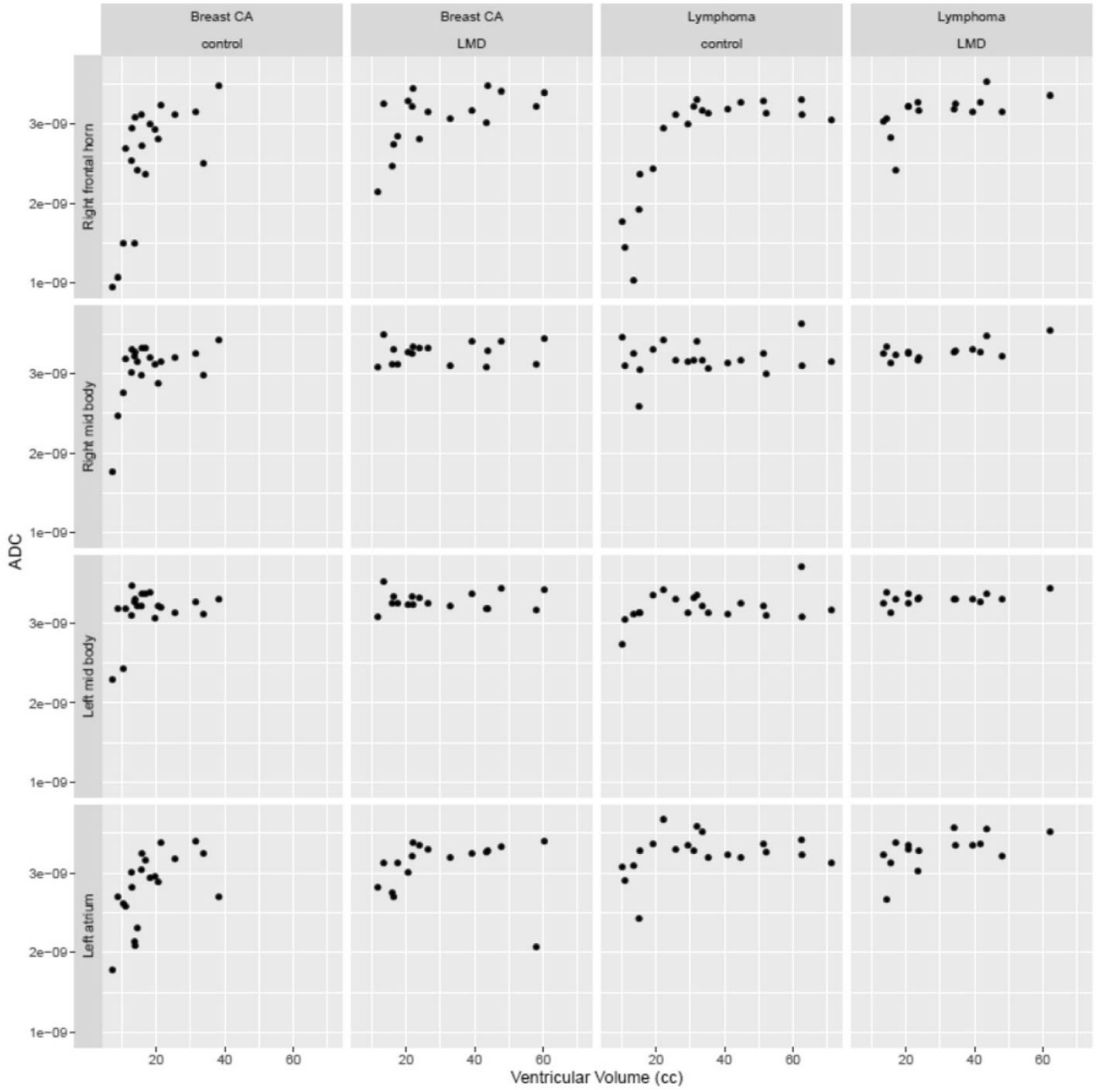
Scattergram of ventricular volume versus ADC values in selected locations. The least fluctuation ADC value across different ventricular volumes was in the right and left mid body of the lateral ventricles

Review of the scatterplots showed that the ADC values had the least ADC value fluctuation across different ventricular size in the right and left mid-body of the lateral ventricles as illustrated in Fig. 3.

### ROC analysis

**Fig. 4:**
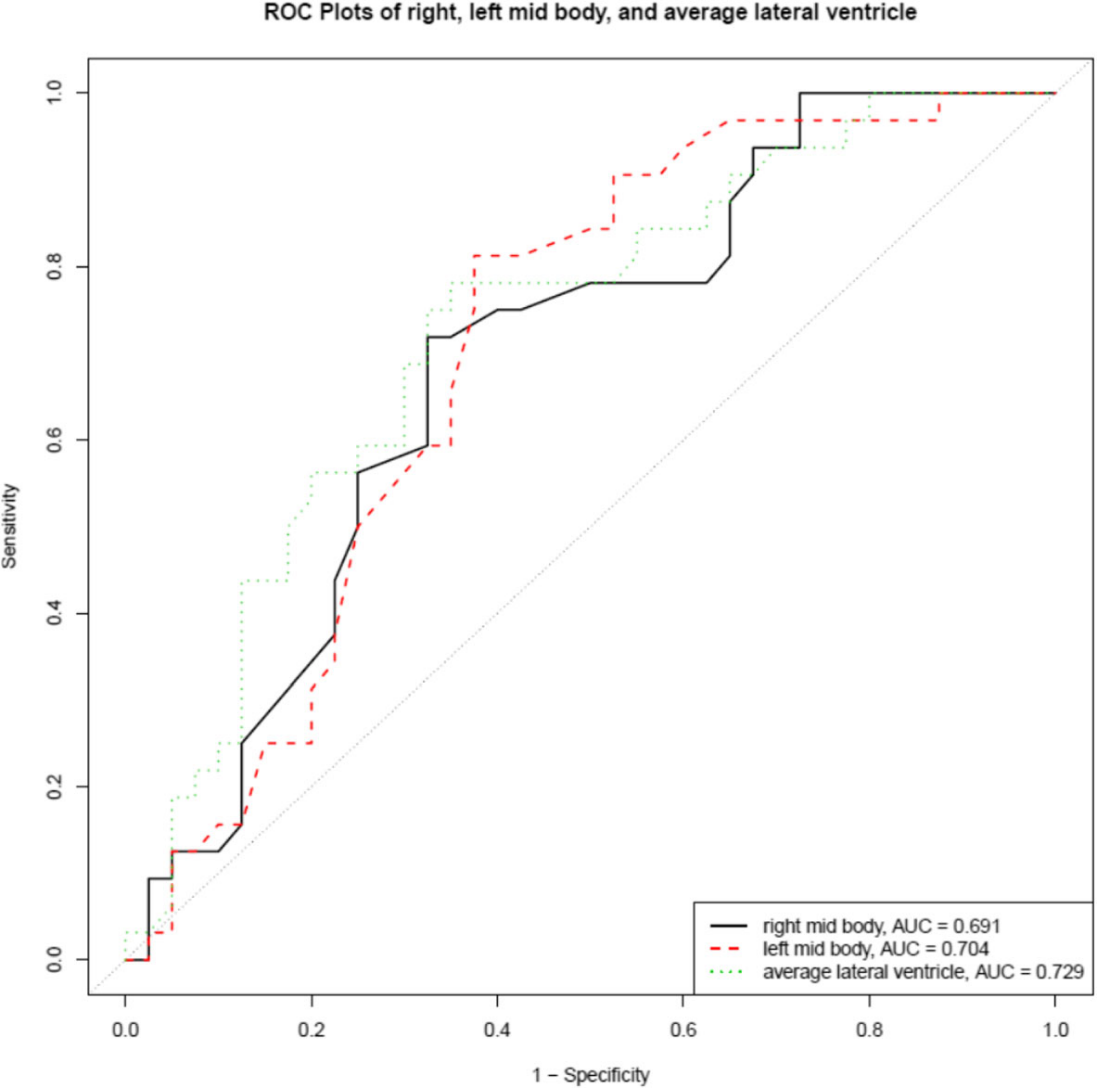
ROC curve from ADC values obtained from the mid body of the lateral ventricles. In the mid body of the right lateral ventricle the AUC was 0.69 (95% CI 0.57–0.81) with an optimal cut off point of 3.22 × 10^− 9^ m^2^/s. In the mid body of left lateral ventricle the AUC was 0.7 (95% CI 0.58–0.82) with an optimal cut off point of 3.23 × 10^− 9^ m^2^/s. Using an average value of HU measurements in the lateral ventricles the AUC was 0.73 (95% CI 0.61–0.84) with an optimal cut off point of 3.11 × 10^− 9^ m^2^/s (0.78, 0.65)

The mid-body of the left lateral ventricles was the only site without a significant difference in ventricular volume that also showed a significant difference in ADC values when patients with LMD were compared to the control groups. As the mid-body of the lateral ventricles also showed the least fluctuation on the scatterplots, ROC analysis was performed on the ADC values measured in this location. In the mid-body of the right lateral ventricle the AUC was 0.69 (95% CI 0.57–0.81) with an optimal cut off point of 3.22 × 10^− 9^ m^2^/s (sensitivity, specificity; 0.72, 0.68). In the mid-body of left lateral ventricle the AUC was 0.7 (95% CI 0.58–0.82) with an optimal cut off point of 3.23 × 10^− 9^ m^2^/s (0.81, 0.62). Using an average value of HU measurements in the lateral ventricles the AUC was 0.73 (95% CI 0.61–0.84) optimal cut off point was 3.11 × 10^− 9^ m^2^/s (0.78, 0.65) (Fig. 4).

### Correlation of T1 post contrast and FLAIR findings with ADC values

A significant difference in the occurrence rate of T1 enhancement (*p* < 0.001) was noted in the patients with LMD and breast cancer (*n* = 16/17, 91.4%) compared to patients with LMD and lymphoma (*n* = 6/15, 40%). Sulcal FLAIR signal hyperintensity in the breast cancer group (*n* = 14/17, 82.4%) and the lymphoma group (*n* = 8/15, 53.3%) were not associated with a significant difference in the rate of occurrence (*p* > 0.08). Multiple logistic regressions to predict the presence of LMD by ADC values after adjusting for the presence of T1 enhancement showed that ADC was predictive of the presence of LMD in the left mid body of the left lateral ventricle (*p* = 0.036). No significant difference in ADC values at the remaining sites in the ventricular system was noted (*p* > 0.05). Multiple logistic regressions to predict the presence of LMD by ADC values after adjusting for the presence of FLAIR signal hyperintensity showed that ADC was not predictive of the presence of LMD at any of the measured sites (p > 0.05).

## Discussion

Our results demonstrated that ADC values in the ventricles in patients with LMD are greater than in a control group without LMD. This finding is the opposite of our hypothesis that the ADC values in the ventricles in patients with LMD would be lower than controls owing to compartmentalization of CSF and suggests relatively freer movement in the LMD group. ROC analysis showed that ADC values measured in the mid-body of the lateral ventricles performed best to predict the presence of LMD. The AUC could be slightly improved by finding an average ADC value by measuring multiple sites in the lateral ventricles. When compared to the T1 post-contrast series the ADC values in the left mid-body of the left lateral ventricle correlated with the presence of LMD.

Complex interactions govern the movement of CSF through the ventricular system. This movement may be similar to Poiseuille’s law for the flow of fluid through a tube, represented by in the following equation:

$$ Q=\frac{\Delta P\pi {r}^4}{8 \eta 1} $$

Where *Q* is the flow rate, Δ*P* is the pressure differential between the two ends, *r* is the radius, Ƞ = is the viscosity of the fluid and *l* is the tube length. The flow rate is directly proportional to the 4th power of the radius of the tube. Thus, an increase in ventricular size, intracranial pressure and CSF viscosity may affect the flow rate.

Other factors affect the movement of CSF through the ventricular system. During systole, CSF flow is cranial to caudal while in diastole the flow reverses from caudal to cranial, filling the lateral ventricles. Overall, there is net caudal flow of CSF related to its production. However, the reversal of flow in diastole induces a complex mixing pattern in the ventricles. The flow of CSF into the spinal canal is related to the gradient between the intracranial compartment [28] and the more compliant nature of the spinal canal. The degree of compliance is believed to be related to the venous plexus, ligamentum flavum and the nerve sheaths [29–31].

Linninger et al. [32] studied CSF flow in the normal brain compared to hydrocephalic brain. They found that in a hydrocephalic brain the peak CSF flow velocity increased by 2.7 times compared to the normal brain. In addition, the larger ventricular dimensions and increased CSF pulsatility increased the rate of volumetric flow by a factor of 10. They also observed that complex CSF flow patterns resulted in stagnant areas and eddy formation in the hydrocephalic brain.

Helenius et al. [33] measured the ADC values in the frontal horn and mid-body of the lateral ventricles in 80 patients including 20 of each in the following age groups: 20–34 years, 35–49 years, and 50–64 years and greater than 65 years old. They found that as patients aged, the ADC values increased and attributed the progressive ADC increase to ventricular enlargement with corresponding increase in turbulent CSF flow. In Helenius’ study the average ADC measurement in entire group of 80 patients in the right and left frontal horns was 2.74 ± 0.27 × 10^− 3^ mm/s^2^ and 2.73 ± 0.27 × 10^− 3^ mm/s^2^, respectively. For patient aged 50–64, which is closest to the mean age of the control groups in our study [breast cancer (52 ± 7.8 years); lymphoma (53.2 ± 20.2 years)], the average ADC values were 2.79 ± 0.5 × 10^− 3^ mm/s^2^ and 2.78 ± 0.25 × 10^− 3^ mm/s^2^. The values of the breast cancer control group in our study were slightly lower in the right frontal horn 2.55 ± 0.73 × 10^− 3^ mm/s^2^ (breast) but comparable on the left 2.79 ± 0.56 × 10^− 3^ mm/s^2^. In the lymphoma control group (mean age 53.2 ± 20.2 years) the average ADC value in the left frontal horn was comparable 2.76 ± 0.69 × 10^− 3^ mm/s^2^ but slightly higher in the left frontal horn 2.93 ± 0.46 × 10^− 3^ mm/s^2^.

In the right and left mid body of the lateral ventricle the average ADC measurements in entire group of 80 patients in Helenius’ study 3.02 ± 0.16 × 10^− 3^ mm/s^2^ on both sides for the entire group and in the 50–64 age group were 3.03 ± 0.15 × 10^− 3^ mm/s^2^ and 3.05 ± 0.15 × 10^− 3^ mm/s^2^ on the right and left, respectively. The ADC values of the breast cancer control group in our study were comparable in the right mid body 3.04 ± 0.38 10^− 3^ mm/s^2^. The ADC values were also slightly higher in the mid body of the right lateral ventricle in the lymphoma control group (3.18 ± 0.22 10^− 3^ mm/s^2^), and also slightly higher in left lateral ventricle in both the breast cancer control groups (3.15 ± 0.29 10^− 3^ mm/s^2^) and the lymphoma control group (3.19 ± 0.19 10^− 3^ mm/s^2^). Differences in Helenius’ study and ours may be related to the small sample size in both groups as well as the post-treatment state with potentially larger size of the ventricles of the control group in our study due to treatment associated volume loss versus normal volunteers in Helenius’ group. Another factor may also be the different position of the ROI in the body of the lateral ventricle in Helenius’ study which was more anterior and closer to the foramen of Monro.

In patients with LMD, other factors may complicate the movement of CSF. Infiltration of tumor into the arachnoid villi, Sylvian fissures base of the brain may impede CSF outflow leading to hydrocephalus and increasing the intracranial pressure [34]. Potentially further complicating the movement of CSF is the adherence of tumor cells to the ventricular walls. For fluid flowing past a rough wall there may be alteration of the heat transference rate via increased turbulence [35]. There may be additional perturbations in flow dynamics of CSF with metastatic deposits within or involving the spinal canal – a consideration we did not evaluate in this study.

In our study, we first compared the ADC values in the control versus study groups at each of the measured sites without adjusting for ventricular size and tumor type. We found that there was a significant difference in ADC values at more sites in the breast cancer group than the lymphoma group. When averaging the ADC values in the lateral ventricles, there was a significant difference between the breast cancer group and control group (*p* = 0.006) while the lymphoma group approached significance (*p* = 0.052). We then compared the ventricular volume measurements in the control versus study groups again without adjusting for ventricular size and tumor type. Our results showed a significant significantly lower volume of the lateral ventricles and total ventricular volume in the breast cancer control group. No significant difference was noted in the volumes in the lymphoma control versus LMD group. This combination of more sites with differences in ADC values between the breast cancer control and LMD group and the significant differences in the ventricular size confirms that a change in ventricular volume may affect the diffusivity of CSF and thus the ADC values.

After adjusting for ventricular volume and tumor type, we found a significant difference in ventricular volume between the control and LMD groups at all sites except for the mid-body of the left lateral ventricles although this site approached a significant difference. Analysis of the scatterplots that compared the adjusted ventricular volume to ADC also showed the least fluctuation in the mid-body of the lateral ventricles. Possible explanations for these findings could include the small sample size and possibly that the lateral ventricular and left atrial volumes were already increased in size in the post-treatment control population or that these sites are less susceptible to change with the presence of active LMD.

When comparing the ADC values between the patients with breast cancer and lymphoma who had LMD, a significant difference was found in the right (*p* < 0.048) and left (*p* < 0.001) atrium and in the third ventricles (*p* < 0.002) with the lymphoma group having a higher ADC values. When comparing ADC values between the control groups and the patients with LMD, a significant difference was noted between the ADC values at all sites except for the left atrium (*p* > 0.09) and fourth ventricle (*p* > 0.068) although these approached a significant difference. We postulate that these findings could be also secondary to sample size or possibly complex interactions of the flow dynamics in the atria, third and fourth ventricles including factors such as an increase flow rate through the foramen of Monro and inherent changes in CSF flow in the ventricles.

In [33] our study, multiple logistic regressions showed that of ADC values in the mid body of the left lateral ventricle were predictive of the presence of LMD. Other sites were not predictive of the presence of LMD, including the right lateral ventricle. This may be related to the significantly higher ADC values noted in the left lateral ventricle in the lymphoma LMD group before correcting for ventricular size and the variation in ventricular size between the breast cancer control and LMD groups. Other contributing factors may also be that the control patients in our study were previously treated for cancer, including radiation therapy that could have altered the ventricular system due to cerebral volume loss or local toxicity. This is demonstrated by the higher ADC values in our control groups compared to the study in normal volunteers by Helenius et al. The relative small sample size may have been another contributing factor.

When measuring the average HU values in the lateral ventricles between the control and LMD groups a significant difference was noted between the ventricular volume and between ADC values. Therefore, further study is necessary to determine the effect of ventricular size on ADC values as governed by Poiseuille’s law. The significantly more common occurrence of T1 enhancement in the breast cancer LMD group than the lymphoma LMD group may have impacted our results. Other limitations include the retrospective single-institutional study and partial volume effects inherent in any ROI measurements. In addition, we used CSF cytology as the gold standard for the presence of LMD. As CSF cytology is only 80–95% sensitive [4] there may be an underestimation of the sensitivity of our results. These factors as well as comparing ventricular ADC values to contrast enhanced FLAIR sequences that are superior to T1 post-contrast sequence for detection of leptomeningeal enhancement [35] could be the subject of future investigations. Further studies could also use artificial intelligence for segmentation of the lateral ventricles or the entire ventricular system to determine if a single ADC value aids in detecting the presence of LMD.

## Conclusion

Complex interactions including ventricular size, flow rate, pressure, CSF viscosity and eddy formation may alter the movement of CSF and affect ADC measurements within the ventricular system in patients with LMD. Elevated ADC values in the lateral ventricles may provide non-invasive clues for the diagnosis of LMD. This study may serve as a baseline to guide future investigations on the use of ventricular ADC values in patients with neoplastic LMD for both validation and refinement before clinical implementation.

## Data Availability

Not applicable.

## Abbreviations

LMD: Leptomeningeal disease
ICC: Intraclass correlation coefficient
SD: Standard deviation
ROC: Receiver operating characteristic
NED: No evidence of disease
